# Evaluating an integrated neighbourhood approach to improve well-being of frail elderly in a Dutch community: a study protocol

**DOI:** 10.1186/1756-0500-4-532

**Published:** 2011-12-13

**Authors:** Jane M Cramm, Hanna van Dijk, Freek Lötters, Job van Exel, Anna P Nieboer

**Affiliations:** 1 Institute of Health Policy and Management, Erasmus University Rotterdam, the Netherlands

**Keywords:** elderly, social network, study protocol, community, neighbourhood

## Abstract

**Background:**

An important condition for independent living is having a well-functioning social network to provide support. An Integrated Neighbourhood Approach (INA) creates a supportive environment for the frail elderly, offering them tailored care in their local context that allows them to improve self-management abilities and well-being. The purpose of our research is to investigate how an INA can contribute to outcomes of frail elderly and the cost-effectiveness of such a program. The first central study question is: To what extent does INA contribute to (a) continuous, demand-driven, coordinated care and support for the independently- living frail elderly; (b) improvement of their well-being and self-management abilities; and (c) reinforcement of their neighbourhood networks. The second central research question is: is the INA a cost-effective method to support the frail, independently- living elderly?

**Methods:**

We investigate a Dutch INA. This transition experiment aims to facilitate the independently-living frail elderly (70+) to live the life they wish to live and improve their well-being. The study population consists of independently-living frail elderly persons in Rotterdam. The transition experiment starts in two Rotterdam districts and is later extended to two other districts. We propose a concurrent mixed methods design, that is, a combination of qualitative and quantitative research methods to evaluate processes, effects and costs of INA. Such a design will provide insight into an on-going INA and demonstrate which of its elements are potentially (cost)-effective for the frail elderly.

**Discussion:**

We embrace a wide range of scientific methodologies to evaluate the INA project and obtain information on mechanisms and contexts that will be valuable for decision making on local and national levels. The study will lead to a better understanding of how to provide support via social networks for the frail elderly and add to the knowledge on the feasibility and cost-effectiveness of the program in maintaining or improving their well-being. Last, the study will highlight the factors that determine the program's success or failure.

## Background

People with highly-functioning social networks are better able to give and receive support, are more psychologically resilient, and live longer and healthier lives [1]. Regrettably, various reports and signals from the field suggest that the current professional approach fails to provide frail elderly people with needed social support networks to make living conditions safer, more stimulating, comfortable, and pleasant and to enable them to live in their own neighbourhoods for a longer time. Strengthening social networks fosters early detection of problems, is crucial to public health, and is expected to reduce the pressure on the healthcare system by preventing or delaying nursing home admissions. Facilitating elderly people through an Integrated Neighbourhood Approach (INA) to live independently for as long as possible requires a supportive community environment, which is in turn dependent on the presence of meeting places [2], mutual interdependence of residents, and motivation to invest in local relationships reflected, for example, by residential stability [3]. Neighbourhood differences in this regard have been reported. An important condition seems to be that the community engage in shared activities, thus establishing contacts through which social networks can develop [4,5]. Residents will be more inclined to participate in neighbourhood activities if they perceive a sense of community [6]. Currently, the frail elderly have to depend on professional care; informal networks and social support are underemployed [7].

The point of departure of INA is reinforcing networks between welfare, health care, informal care and community members in neighbourhoods, optimizing current services, and involving the (frail) elderly. Such a demand-driven approach offers elderly people tailored care - including care-related services such as housing - in their local context to enhance self-management abilities and well-being. The focus is on "de-medicalisation" and recognition of mutual dependence between welfare, health care, and informal care. Thus, for INA to be successful the partners in primary, secondary, and tertiary care as well as informal networks need to work well together - from signalling problems to prevention, cure, care, promotion of welfare, and independent living. Early recognition of complaints and encouraging effective self-management may positively influence well-being. It requires the elderly to 'star' in the 'production' of their own well-being as a form of empowerment [8]. Informal caregivers play a central role in their social networks and are important to supporting independent living. Evidence suggests that caring for a frail elderly person is an arduous task that may cause financial difficulties, emotional strain, or physical problems [9,10]. A supportive network for elderly may alleviate such negative aspects of caregiving, which in turn helps sustain informal caregivers' support.

While INA may improve outcomes, evidence regarding the (cost-) effectiveness of such programmes is lacking. The purpose of our research is to investigate how an INA can contribute to outcomes of the frail elderly and its cost-effectiveness. The first central study question is: To what extent does INA contribute to (a) continuous, demand-driven, coordinated care and support for the independently- living frail elderly and the well-being of their informal caregivers; (b) improvement of their well-being and self-management abilities; and (c) reinforcement of their neighbourhood networks. The second central research question is: is the INA a cost-effective method to support the frail, independently- living elderly?

## Methods

### 

#### Setting: Dutch example of an Integrated Network Approach

Although welfare and health care are widely available in the city of Rotterdam, the specific needs of frail elderly remain inadequately addressed and 'outreach' work is lacking. A number of 'best practices' may exist locally, but not a good overview of the services because of fragmentation and compartmentalisation. Such services are difficult for the elderly to find and are not visible to others in the city. In the current situation the frail elderly have to depend on professional care, while informal networks and social support are underused. An INA is based on reinforcing neighbourhood networks through which continuous, demand-driven, coordinated care and support can eventually be offered to all independently-living frail elderly persons. Community workers - professionals with a care or welfare background familiar with the residential area - are important to the network. They visit the elderly at home and map their wishes and needs via a phased interview. In consultation with the elderly, they seek appropriate solutions within the (preferably informal) network. Such a transition experiment aims to facilitate independently-living frail elderly persons (70+) to live the life they wish to live, improving their well-being. The study population consists of independently-living frail elderly persons and their informal caregivers in Rotterdam. The transition experiment begins in two Rotterdam districts (*Lage Land/Prinsenland *and *Lombardijen) *and is later extended to the *Oude Westen *and *Vreewijk *districts.

The project ('An integrated neighbourhood approach to welfare and care for the frail elderly in Rotterdam') and the associated evaluation study are part of the National Care for the Elderly Programme (NPO) launched in the Netherlands in 2008. Funding is provided by the Netherlands Organisation for Health Research and Development (ZonMw; project number 314030201).

#### Evaluation design

Our evaluation study uses a concurrent mixed-methods design (a combination of qualitative and quantitative research methods) to evaluate processes, effects and costs of INA. A frequent shortcoming of evaluation studies is failure to give good descriptions of what was done and the context in which it was done [11]. In the first phase (months 1-6), therefore, the eventual local-level interventions will be described extensively along with how welfare, care, and network support for frail elderly persons and their informal caregivers is achieved. A good description of interventions is the first step and towards that, key figures including community workers will be interviewed.

The evaluation will comprise (I) inventory and (II) controlled pre-post measurement. Inventory is taken among the elderly (70+) in the four relevant districts (Lage Land/Prinsenland, Lombardijen, Oude Westen, and Vreewijk) to investigate the general situation of elderly in these districts. Furthermore, we investigate social networks, social cohesion, and the sense of community in these districts to learn if INA contributed not only to elderly included in the experiment, but to the wider context as well.

**Figure 1:**
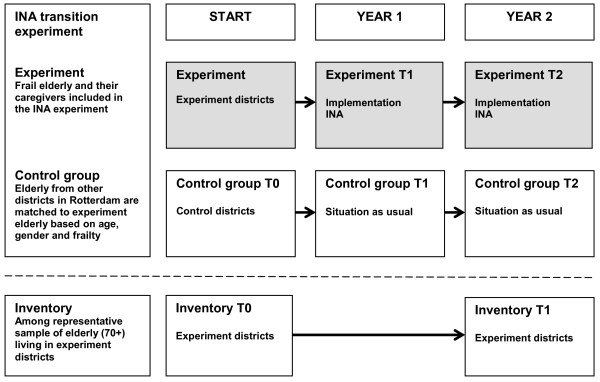
**Study design Integrated Neighborhood Approach**.

The controlled pre-post measurement is the main part of the evaluation. Independently-living elderly (70+) in the first two districts will serve as the experimental group (Figure 1).

#### I. Inventory

A sample of 1440 independently-living elderly (70+) in the four districts will be taken from the population register, 430 eligible elderly per district and proportional to neighbourhood and age. The eligible elderly will be asked by mail to complete a (written or online) questionnaire (T0) whose estimated completion time is 15 minutes. Those who do so will be rewarded with a 1/5 ticket in the monthly Dutch State lottery. Those who do not respond after having been sent a reminder will be telephoned. If not available, they will be visited at home. This strategy is expected to result in a 60% response rate (n = 864). The group will be contacted again after 24 months (T1) to assess whether (i) local social networks have been reinforced, (ii) the elderly participate more actively, and (iii) the frail elderly have built up better personal networks. Using the same strategy as in the T0 measurement (incentives and follow-ups), we expect a 70% response at T1 (which includes a 15% attrition from death, relocation, institutional admission, et cetera), resulting in n = 605.

#### II. Controlled pre-post measurement (effect evaluation)

The independently-living elderly (70+) in the first two districts whose TFI-score is ≥ 5 [12] will serve as the experimental group and will be recruited by community workers. On the basis of TFI-score, age, and gender, they will be matched with the elderly recruited from comparable districts in Rotterdam as a control group. In total we expect to include 370 elderly (247 in the experimental group; 123 in the control group). All will be interviewed at home by experienced interviewers at three time-points: T0, T1 (6 months after inclusion), and T2 (12 months after inclusion). On average the interviews will take 60 minutes.

Informal caregivers will be interviewed twice by telephone for about 15 minutes each. They will be identified on the basis of the definition provided in the National Care for the Elderly Programme: those who provide structured care voluntarily and for free to people in their family, household, or social network with physical, mental or psychological disabilities. It involves providing more care than usual in personal relationship and consists of tasks that healthy people could normally do themselves.

#### Sample size

We will include 370 elderly (2/3 in intervention group, 1/3 in control group). We will try to limit sample losses by personal house visits, but expect a loss of about 27% (by death, moving, no longer wishing to participate, etc.) between T0 and T2, resulting in a final sample of 270. This number - 180 in the intervention group and 90 in the control group - is required to detect a 1-point improvement in TFI-score in the intervention group as compared with the control group at T2 (with mean TFI-score 4.7, sd 3.0; one-sided test; alpha = 0.05, power = 0.80) [12].

##### Ethical approval

The study protocol was approved by the ethics committee **of the Erasmus University Medical Centre of Rotterdam in June 2011. R**espondents will receive a brochure prior to the interview to explain the study and procedure, provide a free helpdesk telephone number, and state that the Medical Ethical Review Board of Erasmus MC has issued a Certificate of No Objection after having established that the study complies with the Dutch Act on Medical Research in Humans. The respondents' informal caregivers will also receive a brochure with information about the study and an invitation to participate. Both the elderly and their informal caregivers will be explicitly informed in the brochure and by the interviewer that participation can end at any time without adverse consequences. Written informed consent will be obtained from all participating respondents.

### Evaluation components

**Figure 2:**
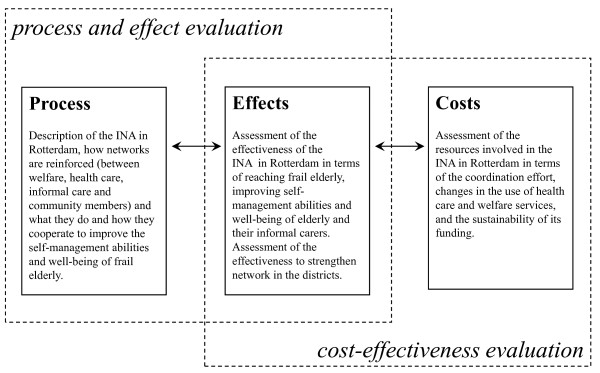
**Process and effect evaluations; cost-effectiveness evaluation**.

The evaluation study has three parts: (i) process (ii) effects, and (iii) costs of INA (Figure 2).

#### i. Process

The process evaluation study will find whether INA contributes to (a) continuous, demand-driven, coordinated care and support for independently-living frail elderly, and (b) reinforcing the welfare, health care, informal care and community networks in their neighbourhoods. We will describe INA in Rotterdam, how the various networks are reinforced, what they do, and how they cooperate to improve the self-management abilities and well-being of frail elderly. Process indicators will be registered continually during 12 months. Data such as descriptions of client visits, assessment outcomes, action goals, and agreements will be captured by a computerized Client Monitoring System and registration forms. An evaluation of the process indicators and data about contacts with professionals is expected to reveal any INA effects.

We will hold semi-structured interviews with professionals, key figures, neighbours, elderly and their caregivers to provide insight into possible barriers and conditions under which proposed changes take place. Earlier research has shown, for example, that conflicting priorities, lack of specificity of and consensus on intended changes, and professionals' insufficient commitment can be important barriers [13]. We will also investigate the experiences of professionals and key figures via questionnaires. Since the effectiveness of a transition experiment is strongly dependent on the implementation process, we would like to know what conditions promote or limit the effectiveness of welfare and care support in neighbourhoods to get an even better understanding of the success of the intervention(s) and the merit of INA for other settings [11]. All professionals (community workers, district nurses) and key figures directly involved in the care and support of the elderly will be given a written questionnaire at T0 and T1. The instrument is partly based on the partnership self-assessment tool [14], which is currently being tested in a disease management study [15] and validated via interviews in its first phase. Aspects addressed in the questionnaire are (a) participation of the professionals and key figures involved in INA (partnership synergy); (b) different dimensions of partnership functioning (leadership, control and management, efficiency, non-financial resources, challenges in partners' commitment and to the municipality/district); and (c) relational coordination (frequency of communication between parties involved, quality of the communication, extent of shared goals, knowledge, and respect) [16] (table 1).

**Table 1:** Outcome and process instruments

Primary outcomes elderly	Instruments	Items
**Frailty**		
Tilburg Frail Indicator (TFI)	Questionnaire	15 items
**Quality of life**		
Short Form 20 (SF-20)	Questionnaire	20 items
EuroQol (EQ-6D)	Questionnaire	6 items
Visual Analogue Scale (VAS)	Questionnaire	1 item
Social Production Function Instrument for Level of wellbeing (SPF-IL)	Questionnaire	15 items

**Secondary outcomes elderly**	**Instruments**	**Items**

**Health outcomes, functioning and abilities**		
Cognitive functioning	Questionnaire	6 items
Katz Index of Independence in Activities of Daily Living (ADL)	Questionnaire	15 items
Self Management Ability Scale Short version (SMAS-S)	Questionnaire	18 items
**Health behavior**		
Smoking behavior	Questionnaire	3 items
Physical Activity	Questionnaire	1 item
**Health care utilization**		
Health care utilization	Questionnaire	18 items
**Neighborhood experiences**		
Social cohesion and belonging	Questionnaire	15 items
Neighborhood quality index	Questionnaire	15 items
**Social resources**		
Social support index	Questionnaire	20 items
Social connection index	Questionnaire	5 items
Social support of partner/children/family and friends/neighbors	Questionnaire	6 items
Social capital	Questionnaire	9 items
Social participation	Questionnaire	2 items

**Outcomes caregivers**		

**Quality of life**		
Short Form 20 (SF-20)	Questionnaire	20 items
CarerQoL-7D	Questionnaire	7 items
CarerQol-VAS	Questionnaire	1 item
Social Production Function Instrument for Level of wellbeing (SPF-IL)	Questionnaire	15 items
**Health outcomes**		
Katz Index of Independence in Activities of Daily Living (ADL)	Questionnaire	15 items
**Caregiving experiences**		
Activity restriction scale	Questionnaire	10 items
Caregiver Strain Index (CSI+)	Questionnaire	18 items
Self-Rated Burden	Questionnaire	1 items
**Health care utilization**		
Health care utilization	Questionnaire	14 items
**Social resources**		
Social support of partner/children/family and friends	Questionnaire	6 items

**Process outcomes**		

Partnership Self-Assessment Tool Short version (PSAT-S)	Questionnaire	24 items
Relational Coordination Survey	Questionnaire	7 items
Intervention and other direct costs	Data registration	

#### ii. Effects

Assessment of effectiveness will be in terms of reaching the frail elderly, improving their self-management abilities and well-being, and improving the well-being of their informal carers. Demographic data and outcome indicators - well-being, quality of life, self-management abilities, cognitive functioning, social networks, social cohesion, sense of community in the neighbourhood - will be captured with specific instruments (table 1).

#### Instruments elderly

##### Frailty

The Tilburg Frailty Indicator (TFI) will be used to measure frailty. The results regarding the TFI's validity provide strong evidence for an integral definition of frailty consisting of physical, psychological, and social domains [12].

##### Quality of life and well-being

The Dutch version of the SF-20 is administered to the frail elderly. It aims to score 6 sub-dimensions such as physical functioning, social functioning and experienced health [17,18]. The SF-20 was chosen over the SF-36 because it is shorter and because many questions are included in the MDS. The EuroQol (EQ6D) and Visual Analogue Scale (VAS-scale) - part of the MDS - are administered to measure quality of life among the elderly and their informal caregivers. They will also be used to calculate cost-utilities of health care [19].

##### Well-being

The Social Production Function Instrument for the Level of wellbeing scale (SPF-IL) is used to measure the universal goals needed to be realized by individuals in order to enhance their well-being [20]. Social production function (SPF) theory asserts that the universal goals affection, behavioural confirmation, status, comfort and stimulation are the relevant dimensions of subjective well-being. Examples of questions are: 'do you feel that people really love you' and 'are you known for the things you have accomplished'.

##### Self-reported cognitive function

The MOS cognitive function scale will serve as the self-report measure of cognitive function. This scale contains six Likert-type items on memory, reasoning and thinking. The responses to individual questions are summed and the score is then converted to a 0-100 point scale, with 100 indicating the most favorable functioning [21,22]. Examples of items are: 'How much of the time during the past month did you have difficulty reasoning and solving problems, for example making plans, making decisions or learning new things' and 'How much of the time during the past month did you have trouble keeping your attention on any activity for long'.

##### Physical functioning

The Katz-15 index of activities of daily living measures function over time by means of statements on several domains such as bathing, dressing, toileting, transferring, continence and feeding [23]. Example: 'Moves in and out of bed or chair unassisted. Mechanical transferring aides are acceptable' or 'Needs help in moving from bed to chair or requires a complete transfer'.

##### Self-management

The SMAS-S (Self Management Ability Scale-Short version) measures a person's ability to manage his/her own general daily life activities in the past months. It contains 18 items on several self-management abilities [24,25]. Examples are: 'How often do you take the initiative to keep yourself busy?' and 'Are you capable of taking good care of yourself?'

##### Social cohesion & belonging

The neighborhood cohesion scale consists of 15 items on a person's contribution to the social cohesion in the neighborhood [26]. Examples are: 'I would be willing to work together with others on something to improve my neighborhood' and 'I regularly stop and talk with people in my neighborhood'.

##### Neighborhood quality

The Neighborhood Quality Index will be used to capture residents' perceptions of neighborhood quality [27]. Examples are: 'participating in activities together' and 'feeling safe in this neighborhood'.

##### Social support

The social support index will be used to assess levels of social support. This survey was designed to be comprehensive in terms of recent thinking about the various dimensions of social support. Multitrait scaling analyses supported the dimensionality of four functional support scales (emotional/informational, tangible, affectionate, and positive social interaction) and the construction of an overall functional social support index [28].

##### Social connections

The social connections index will be used to assess the level of social connections. This index contains five questions regarding social connections and has shown to be a predictive tool of mortality [29].

##### Social support of spouse, children, friends and relatives, and neighbors

This instruments assesses emotional support, instrumental support and negative aspects of relationships. Example of emotional support is 'how often does/do your [spouse/children/friends and relatives/neighbors] make you feel loved and cared for?" Example of instrumental support is 'how often does/do your [spouse/children/friends and relatives/neighbors] give you advice or information about medical, financial, or family problems?" Negative aspects of relationships were measured by two items that assessed the frequency with which participants' spouses, children, friends and relatives, or neighbors 'made too many demands' or 'were critical' [30].

##### Social capital

The Short Social Capital Assessment Tool (SASCAT) serves to assess social capital. The tool could also be used to measure ecological social capital by administering it to a representative sample of a community and aggregating their responses [31]. Examples of items are: 'in the last 12 months, did you receive from the group any emotional help, economic help, or assistance in helping you know or do things' and 'in general, can the majority of people in this community be trusted'.

##### Social participation

Following the study of Guillen and colleagues [32] we will measure social participation with the following questions: 'compared to other people of your age, how often would you say you take part in social activities' and 'how often do you meet socially with friends, relatives or neighbors'.

#### Instruments caregivers

##### Quality of life

In addition to the instruments also used with the elderly, the carer quality of life questionnaire (Carer QoL-7D) measures quality of life of informal carers and is part of the MDS [33,34].

##### Activity restriction

Burden of care for the carer is measured using the Activity Restriction Scale (ARS) [35]. Carers are asked to indicate the extent to which nine areas of normal activity (e.g., doing household chores, going shopping, visiting friends, participating in sports and recreation, maintaining friendships) are restricted by their caregiving responsibilities.

##### Self-rated burden and strain

Subjective burden of care is measured with the Self Rated Burden Scale (SRBS) and Caregiver Strain Index or CSI [36,37]. Examples of questions are: 'there have been family adjustments (e.g. helping has disrupted my routine; there is no privacy)' and 'there have been changes in personal plans (e.g. I had to turn down a job; I could not go on vacation)'.

#### iii. Costs

A cost-effectiveness evaluation will be performed to determine whether INA is a cost-effective method to support the frail, independently-living elderly. We will assess the additional (health care) costs involved in INA and the costs per quality-adjusted life years (QALY) gained in the elderly and their informal caregivers. INA costs may be higher than expected because extra care and support are offered and more people could avail themselves of the services, or they may be lower than expected because specific groups of elderly and their informal caregivers will earlier and more purposefully avail themselves of the services and receive better support from their networks, preventing or delaying serious (health) problems. Delaying or preventing admission to a nursing home, for example, lowers costs and often appeals to the elderly and informal caregivers.

Health care utilization and given support will be quantified via questionnaires and additional sources where possible (Client Monitoring System, local and national monitors). Multiplying these volumes by integral cost prices will yield total costs of care and support. For this purpose we will use the guideline of the Dutch Health Care Insurance Board (CVZ) [38]. INA costs are estimated via time registrations on professionals' activity levels. Different types of activity (such as contact with the elderly and team meetings), professional disciplines, and corresponding tariffs will be taken into account. Assessment costs will be included in the total costs for the intervention group only and not the control group, because the costs are incurred only within the INA framework. Finally, costs per centre will be calculated (e.g., costs of the interventions, welfare, health care, community workers), providing insight for all participating organizations as to the investments that will be needed to continue INA after the study phase.

The cost-effectiveness evaluation will be on the basis of the costs and the registered effects described above. The primary analysis is a regular cost-utility analysis with differences between the intervention and control groups in costs and well-being (QALY) during a 12-month follow-up as outcome, allowing us to compare findings with other studies.

### Data analysis

Data on defined outcome measures for the process- and (cost-)effectiveness evaluations will be collected at T0, T1, and T2 for the elderly and at T0 and T2 for the informal caregivers. They will be described and analysed as follows:

- Descriptive statistics at the group and district levels at different time points;

- Bivariate analyses relating outcome measures to the elderly's socio-demographic characteristics and process indicators;

- Correlation analysis between various types of outcomes;

- Multivariate analysis of outcome measures per time point and longitudinally;

- Subgroup analyses to determine whether outcomes strongly vary for different groups (e.g., single vs. partnered, low vs. high self-management abilities);

- Sensitivity analyses to determine the influence of major assumptions on reported outcomes.

### Integration of findings

Methodologically, the assessment of a transition experiment comprises the evaluation of a complex mixture of interventions at the elderly, professional, and non-professional levels. Qualitative and quantitative methods are used to answer the same research questions and are thus mixed throughout all project phases, from the design stage to data interpretation. The method enables us to understand (i) the mechanisms through which changes are produced, (ii) the contextual conditions necessary to trigger such mechanisms, and (iii) the effects of interventions with respect to context and triggered mechanisms. Intermediate results of the qualitative, quantitative, and cost-effectiveness analyses will be continually looped within the research group to allow for improvements and recognition of emerging themes across research methods and a more fine-grained data analysis. This is especially relevant for the qualitative component of the project. Although different researchers will have responsibility for different parts of the study, regular team interaction will ensure optimal integration of results.

## Discussion

To describe effects of INA we will use a methodological approach that combines qualitative and quantitative research. Introducing complex, multi-component interventions is sensitive to an array of influences such as details of implementation and context [13,15] and calls for embracing a wide range of methodologies to obtain information on both mechanisms and contexts, add to knowledge on the approach's feasibility and costs, and highlight the factors likely to bring success or failure. While descriptive studies may provide appropriate understanding of mechanisms and context of change, they lack rigor in terms of understanding the intervention's effectiveness.

### Weaknesses

In our study, health care utilization is mainly derived from questionnaires administered to the frail elderly and their informal caregivers instead of using direct and perhaps more accurate information from health care companies. Unfortunately, there is a long delay in declaration and registration of health care costs, which hampers the timely delivery of the information needed for the cost-effectiveness evaluation. Moreover, extracting information from the database of the healthcare insurance companies requires obtaining informed consent to collect the additional data in addition to written informed consent to participate, perhaps decreasing participation. Another drawback in using questionnaires might be the recall bias for health care utilization over the past three to six months, but the questionnaires will be administered by means of face-to-face interviews with the frail elderly, giving the interviewer opportunity to ask for clarification. And while we are not able to randomize the frail elderly in the intervention and control groups; we will match the two groups.

### Strengths

We will embrace a wide range of scientific methodologies to evaluate the INA project and obtain information on mechanisms and contexts that will be valuable for decision making on local and national levels. The study will thus lead to a good understanding of the mechanisms providing social network support for frail elderly and add to the knowledge on its feasibility and cost-effectiveness in maintaining or improving well-being. Furthermore, the study will highlight the factors that determine the success or failure of such programs.

Implementation of large interventions within Dutch municipalities is not often accompanied by a thorough cost-effectiveness evaluation from a societal perspective. It enables us to give a sound description of the costs of the INA intervention and benefits from the perspective of different stakeholders (i.e., the elderly, the municipality, caregivers, and health insurers).

## Competing interests

The authors declare that they have no competing interests.

## Authors' contributions

JC, AN, and JvE participated in the study design. JC and AN drafted the manuscript. HvD, FL, and JvE helped drafting the manuscript and contributed to its refinement. All authors have read and approved the final version.
